# An Opioid-free Anesthesia Protocol for Pediatric Strabismus Surgery: A Quality Improvement Project

**DOI:** 10.1097/pq9.0000000000000462

**Published:** 2021-08-26

**Authors:** Jennifer L. Chiem, Laura D. Donohue, Lynn D. Martin, Daniel K. Low

**Affiliations:** From the Department of Anesthesiology and Pain Medicine. Seattle Children’s Hospital. University of Washington, Seattle, Wash.

## Abstract

**Introduction:**

This quality improvement (QI) project tracks a series of 2 Plan-Do-Study-Act (PDSA) cycles as we standardized and refined an ambulatory pediatric anesthesia strabismus protocol. We aimed to provide effective pain relief, reduce postoperative nausea and vomiting (PONV) rates, and be cost-efficient while minimizing perioperative opioids over 5 years.

**Methods:**

We used statistical process control (SPC) charts to analyze real-world data captured from the medical record. We chose the following outcome and process measures to evaluate effectiveness: postoperative morphine rescue rate, maximum pain score in the postanesthesia care unit (PACU), and PONV rescue rate. We also used 2 balancing measures: postoperative length of stay (LOS) and total anesthesia time. We standardized our anesthesia protocol for our first PDSA cycle (April 2017) by removing intraoperative intravenous acetaminophen and utilizing fentanyl only. For the second PDSA cycle (January 2019), we replaced intraoperative fentanyl with dexmedetomidine.

**Results:**

There was a total of 325 pediatric strabismus repair surgeries performed between April 2015 and July 2020. There was no special cause variation detected in the SPC charts for the family of measures chosen to measure effectiveness: postoperative morphine rescue rate, maximum pain score in the PACU, or the PONV rescue rate. The PONV rescue rate was 0 with the removal of opioids. Also, there was no special cause variation for the balancing measures: postoperative LOS or total anesthesia time.

**Conclusions:**

Throughout 2 PDSA cycles, this QI project enabled our team to standardize an opioid-free and cost-efficient anesthesia protocol for pediatric strabismus surgery over 5 years.

## INTRODUCTION

Strabismus surgery is a common pediatric ophthalmologic surgery. Risk factors for strabismus include prematurity, low birth weight, and a history of retinopathy of prematurity.^1–4^ Anesthetic concerns include oculocardiac reflex, postoperative nausea and vomiting (PONV), emergence delirium (ED), and postoperative pain control. Analgesia is typically achieved with intravenous (IV) opioids. Administration of dexamethasone, ondansetron, and liberal IV fluids are common prophylaxis measures for nausea and vomiting.^5^ Treatment of ED includes benzodiazepines and dexmedetomidine.^6^

At Seattle Children’s Bellevue Clinic and Surgical Center (BCSC), approximately 90 strabismus repairs are performed annually. Due to the opioid shortage in 2017–2018, BCSC anesthesiologists explored methods to conserve the opioid supply by pivoting to opioid-sparing pain management strategies. One methodology utilized dexmedetomidine, a highly selective alpha-2-agonist with analgesic and anxiolytic properties but without the respiratory depressant effects of opioids.^7^ The use of dexmedetomidine in the current pediatric literature focuses upon treatment of ED,^8–10^ sedation for the intensive care unit,^10,11^ and preoperative anxiety.^12^ A tonsillectomy and adenoidectomy (T&A) quality improvement (QI) project at BCSC demonstrated similar pain scores and postanesthesia care unit (PACU) length of stay (LOS) when utilizing dexmedetomidine and ketorolac as compared to morphine and acetaminophen, as well as improved PONV rescue rates.^13^ Based upon the T&A QI project findings, the BCSC facility expanded its protocols to utilize dexmedetomidine, which minimizes opioids’ use for all its pediatric ambulatory surgeries.^14^ The expansion of dexmedetomidine use was implemented in a phased manner to track its effectiveness.

We aimed to standardize an anesthetic protocol to optimize pain management and PONV in a multimodal approach for pediatric strabismus surgery over 5 years. We selected maximum PACU pain score, postoperative morphine rescue rate, and PONV rescue rate as primary outcome measures.

## METHODS

BCSC is an ambulatory outpatient facility where clinicians incorporate standardized clinical pathways into their practice. Anesthesiologists and certified registered nurse anesthetists can track the adoption and effectiveness of protocol changes due to the healthy patient population, low acuity, high-volume caseload, and the informatics infrastructure built around real-world electronic medical record (EMR) data starting in 2015.

In this QI project, we included the American Society of Anesthesiologists class 1–3, patients 1–17 years old of age undergoing strabismus surgery at BCSC from April 1, 2015, through July 30, 2020. We chose this time frame due to the variability of strabismus cases performed and to encourage adherence to multiple concurrent protocol changes at the BCSC facility.

Since 2010, the anesthesia protocol for strabismus surgery included the following: fentanyl IV (1–2 µg/kg) and ketorolac IV (0.5 mg/kg) for pain control, and dexamethasone IV (0.15 mg/kg), ondansetron IV (0.15 mg/kg), and IV fluids (20 mL/kg) for PONV (Table 1). This protocol is incorporated within our anesthesia EMR, which is updated when changes are made to the protocol. Upon reviewing the real-world data for the strabismus protocol, there was variability in the intraoperative administration of acetaminophen IV (cohort A/F). Only 26% of strabismus cases received it. However, 98% of strabismus cases received intraoperative fentanyl. The average wholesale cost of acetaminophen IV is $40 per vial, whereas fentanyl IV is $2 per vial. Thus, we removed intraoperative intravenous acetaminophen for our first Plan-Do-Study-Act (PDSA) cycle intervention on April 1, 2017. Oral acetaminophen was available for postoperative pain control in the PACU, if needed. This change was a cost-reduction as well as a standardization effort. Intraoperative fentanyl was the primary analgesic medication administered (cohort F). This intervention change was announced during our monthly anesthesia morning conference as well as updated in the EMR. From April 1, 2017, to January 1, 2019, 18% of strabismus cases still received intraoperative acetaminophen, whereas 90% of strabismus cases received intraoperative fentanyl.

**Table 1.** Anesthesia Protocols

Acetaminophen/Fentanyl (A/F) Cohort N = 82	Fentanyl (F) Only Cohort N = 130	Dexmedetomidine (D) Only Cohort N = 113
April 1, 2015–March 31, 2017	April 1, 2017–December 31, 2018	January 1, 2019–July 1, /2020
Induction—Sevoflurane 8%/Oxygen/Nitrous Oxide Propofol 1–2 mg/kg IV for LMA placement Maintenance—Sevoflurane 0.8–1.2 MAC/<30% oxygen/air Dexamethasone 0.15 mg/kg IV (maximum 4 mg) Ondansetron 0.15 mg/kg IV (maximum 4 mg) Lactated Ringers 20 mL/kg IV Ketorolac 0.5 mg/kg IV (max 30 mg) once surgery was complete		
Acetaminophen IV 15 mg/kg IV (max 1 g) intraoperatively Fentanyl 1–2 µg/kg IV	Fentanyl 1–2 µg/kg IV	Dexmedetomidine 1 µg/kg IV bolus at induction

MAC, minimum alveolar concentration.

On January 1, 2019, we made our second PDSA cycle intervention, in which dexmedetomidine (1 µg/kg) (cohort D) replaced fentanyl IV (1–2 µg/kg) in the strabismus protocol. The average wholesale cost of dexmedetomidine IV is $17 per vial. However, the BCSC pharmacy can partition a single dexmedetomidine vial into 10 single-use syringes, thereby decreasing the cost to approximately $2 per syringe, which is equivalent to 1 vial of fentanyl. From January 1, 2019, to July 30, 2020, 2% of strabismus cases received intraoperative acetaminophen, 5% of strabismus cases received intraoperative fentanyl, and 99% of strabismus cases received intraoperative dexmedetomidine.

The PACU maximum pain score was a primary outcome measure used to assess the effectiveness of the different protocols. The PACU nurses record pain scores using assessment tools at their discretion. For patients 1–3 years old, the nurses use the Faces, Legs, Activity, Cry, Consolability scale (FLACC; validity r= 0.41–0.8, reliability 61%–91%, kappa = 0.52–0.82). For patients 3–6 years old, the nurses use the Faces Pain Scale-Revised (FPS-R; validity = 0.84–0.99, inter-rater correlations = 0.84–0.99). The nurses use the numerical 0–10 visual analog scale (validity = 0.61–0.90, reliability 0.41–0.58, inter-rater correlation 0.28–0.72) for patients 7 years old and older.^15,16^ We converted each pain assessment tool into an 11-point (0–10) score for the analyses.

Another primary outcome measure was the postoperative morphine rescue rate. PACU nurses administer oral acetaminophen for mild pain (score 1–3). Morphine is the first-line rescue analgesia for moderate (score 4–6) to severe (score 7–10) pain in the PACU.

The PONV rescue rate was an additional outcome measure. Nurses administer PONV rescue medications if the patient is vomiting or if the patient or caregiver notes that the patient is feeling nauseated.

We studied PACU LOS and total anesthesia time as balancing measures. Dexmedetomidine can delay arousal and increase time to discharge,^17^ which is not preferable in a high turnover ambulatory surgical center. Patients and families were discharged directly from PACU to home once they returned to their preoperative baseline or met discharge criteria based upon the Aldrete scoring system.^18^

MDmetrix OR Advisor (MDmetrix, Seattle) is a software system that extracts continuously updated, aggregated, and de-identified health information from the hospital’s EMR. It then presents the data as statistical process control (SPC) charts.^13,19,20^ SPC methodology enables clinicians to distinguish between common cause variation (random variation intrinsic to the system), from nonrandom variation or special cause variation (SCV), such as improvements in protocol changes monitoring a process over time and combining sequential, time-based analyses.^19^ The goal is to see either no SCV (interventions did not worsen postoperative pain score or increase PONV) or a change indicating improvement.

In this QI project, we used 2 types of SPC charts. The P-chart displays dichotomous data as a percentage or proportion of the total data count.^19,20^ We utilized a P-chart for postoperative rescue morphine administration in the PACU. An X-bar chart displays continuous data for subgroups with more than one data value per subgroup.^19,20^ We utilized an X-bar chart for maximum pain score, PACU LOS, and total anesthesia time. We set the upper and lower control limits at three sigmas above and below the mean, respectively. We identified SCV in the case of the following criteria: (1) a single point located outside the control limits; (2) a run of 8 or more points in a row above or below the mean centerline; or (3) 2 out of 3 consecutive points located near the outer one-third of the control limit. A run of 12 points without SCV is representative of a stable process.^19^

We submitted this QI project to the Seattle Children’s Institutional Board Review. The board did not deem it as a research study, and there was no further review.

## RESULTS

**Fig. 1.**
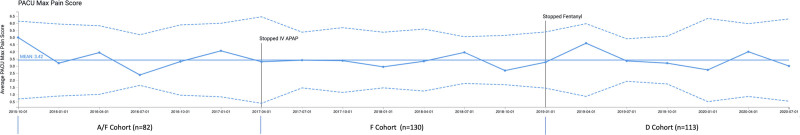
Maximum PACU pain score. The dashed lines above and below the means represent the upper and lower control limits, respectively. APAP, acetaminophen; PACU, postanesthesia care unit.

**Fig. 2.**
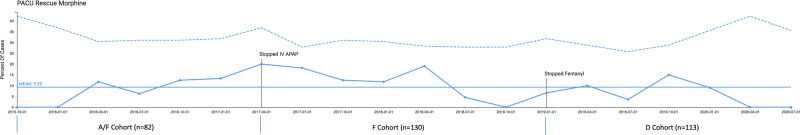
Post-operative morphine rescue rate.

**Fig. 3.**
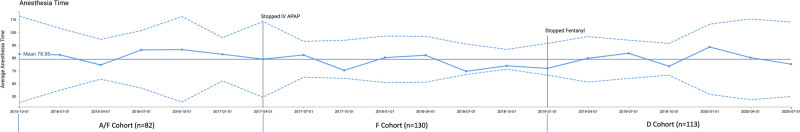
Total anesthesia time.

**Fig. 4.**
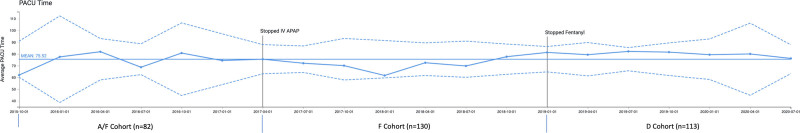
PACU length of stay.

There were 82 patients in the acetaminophen/fentanyl (A/F) cohort, 130 patients in the fentanyl (F) cohort, and 113 patients in the dexmedetomidine (D) cohort. Table 1 shows how the anesthesia strabismus protocol changed over time. Table 2 lists demographic data for the individual cohorts. Figures 1–4 list the SPC results.

**Table 2.** Patient Demographics

Cohort	Acetaminophen/Fentanyl, n = 82	Fentanyl, n = 130	Dexmedetomidine, n = 113
Sex (n/%)			
Male	48 (58.5%)	62 (47.7%)	52 (46.0%)
Female	34 (41.5)	68 (52.3%)	61 (54.0%)
Age, y (mean/range)	5.3 (1–16)	5.8 (1–17)	6.3 (1–17)
BMI, kg/m^2^ (mean/range)	17.3 (13.0–31.5)	18.2 (13.5–36.0)	18.2 (13.0–43.0)
ASA score (n / %)			
1	30 (37%)	62 (47.7%)	43 (38.1%)
2	51 (63%)	66 (50.8%)	63 (55.8%)
3		2 (1.5%)	7 (6.2%)
Race (n/%)			
White or Caucasian	46 (56.1%)	78 (60.0%)	64 (56.6%)
Black or African American	1 (1.2%)	6 (4.6%)	6 (5.3%)
Asian	8 (9.8%)	15 (11.5%)	7 (6.2%)
Other	16 (19.5%)	24 (18.5%)	20 (17.7%)
Patient refused	11 (13.4%)	7 (5.4%)	16 (14.2%)

ASA, American Society of Anesthesiologists Classification; BMI, body mass index.

The X-bar chart in Figure 1 shows the maximum PACU pain score. The chart shows a centerline mean of 3.42. We annotated the 2 PDSA interventions, and the measure is stable throughout both PDSA cycles (no SCV signals detected).

Figure 2 shows the P-chart for the mean postoperative morphine rescue rate. Morphine is our first-line medication used to treat moderate (4–6) and severe (7–10) pain. The centerline mean indicates 9.29% of patients required morphine in PACU. This process remained stable throughout both PDSA cycles (no SCV signals detected).

Only three patients required a PONV rescue medication (1 in the A/F cohort, 2 in the F cohort). No patients required PONV rescue medication in the opioid-free protocol (D cohort).

Figure 3 depicts the X-bar chart for total anesthesia time. Anesthesia start time begins with induction and ends after the anesthesia provider hands off to the PACU nurse. The centerline mean is 79 minutes and did not show any SCV signals.

Figure 4 illustrates the X-bar chart for PACU LOS. PACU LOS begins with the handoff from an anesthesia provider postoperatively and ends with patient discharge from PACU to home. The centerline mean is 76 minutes and did not show any SCV signals.

## DISCUSSION

This project consisted of 2 PDSA cycles, assessing the comparative effectiveness of three protocols utilizing intravenous acetaminophen, fentanyl, and/or dexmedetomidine over 5 years.

We did not see a notable change in PACU maximum pain scores, morphine rescue requirements, PONV rescue medication rates, or LOS in all 3 protocols. Most importantly, the final protocol successfully reduced opioid use without increased pain scores and achieved a zero PONV rescue rate. This information can help clinicians tailor an effective and cost-efficient protocol for pediatric patients undergoing strabismus surgery.

The first PDSA intervention of removing IV acetaminophen from anesthesia protocols (protocol F), primarily as a cost-reduction strategy (estimated annual savings were approximately $10,000 per year), demonstrated no detectable change in PACU maximum pain score or morphine rescue rates. Additionally, there was no increase in PONV or PACU LOS. It is important to weigh the risk and benefit of each medication, including the cost of medications. A case-by-case administration of IV acetaminophen may be more appropriate since its addition did not alter morphine rescue rates. Evidence-based care plans are crucial for establishing best practices.

The second PDSA intervention of removing fentanyl and adding dexmedetomidine (protocol D) showed similar results. PACU maximum pain scores, morphine rescue rates, PONV rates, and LOS were all unchanged. Postoperative pain from strabismus surgery has been reported as high as 65%.^21,22^ Administration of opioids for pain control is not without consequence. Common side effects include nausea, vomiting, itching, and potential respiratory depression. Our goal of effective discontinuation of opioids while maintaining low rates of moderate-to-severe pain is a crucial component in determining an effective anesthesia protocol’s viability. Dexmedetomidine has been well established as an analgesic adjunct and can reduce the need for opioids.^13,23^ This study demonstrated similar results. From a cost analysis perspective, the use of one vial of fentanyl is similar to one pharmacy partitioned syringe of dexmedetomidine at our facility.

Strabismus surgery is highly emetogenic, with PONV rates ranging from 30% to 80%.^24^ Previous studies have suggested that dexmedetomidine can reduce PONV.^14,25^ We used PONV rescue administration in the PACU as a surrogate for PONV rates. This study showed no change in PONV rescue medication requirements with A/F, F, or D protocols. Our historical PONV rates, with the administration of two prophylactic antiemetic medications, were already very low (<1%). This finding would explain why we did not detect a further reduction. In the opioid-free group (protocol D), the PONV rate was zero. Removing opioids and reducing an additional risk factor for PONV is very beneficial in high-risk patients. PONV increases PACU LOS; it also correlates with patient satisfaction and is an essential component of patient care plans.

Dexmedetomidine has the potential to prolong emergence and LOS in the perioperative period. The stability in our total anesthesia time and PACU LOS X-bar chart indicated no deterioration of this measure. The utilization of dexmedetomidine is common in several other protocols at BCSC. Therefore, we speculate that practitioners are already comfortable with the use and pharmacokinetics of this medication. This possibility may account for the lack of significant change in these measured outcomes.

This study did have limitations. We did not isolate the effect of acetaminophen on pain control. In the initial protocol (A/F), only 26% of patients received acetaminophen. The fentanyl group (F) had 18% of cases still receiving intraoperative acetaminophen. Removal of acetaminophen from the protocols did not produce a change in pain scores or morphine use rates. At the time of the study, the focus was to remove opioids safely and decrease patients’ costs. A future PDSA intervention worth exploring is the administration of preoperative oral acetaminophen and intraoperative dexmedetomidine.

We screen the patient population for BCSC to ensure the appropriateness of outpatient surgery, and thus, it is a predominantly American Society Of Anesthesiologists class 1 and 2 pediatric population. We are not attempting to make gross generalizations about populations outside the facility. Recommendations should be considered for appropriate patient populations.

## CONCLUDING SUMMARY

Through QI methodology, leveraging PDSA cycles and SPC charts, we optimized an anesthesia protocol for strabismus surgery that eliminated the need for intraoperative opioids without increasing postoperative morphine requirements and achieved zero PONV rescue medication administration. We present our methods of using real-world data to improve our pediatric population’s outcome measures and successfully established an opioid-free, nausea-free protocol for strabismus surgery not previously published.

## DISCLOSURE

Dr. Low is the Chief Medical Officer and founder of MDmetrix, and Dr. Martin is a shareholder in MDmetrix. The other authors have no financial interest to declare in relation to the content of this article.
